# Hydrogen bonding in 2-carboxy­anilinium dihydrogen phosphite at 100 K

**DOI:** 10.1107/S1600536809007077

**Published:** 2009-03-06

**Authors:** Nourredine Benali-Cherif, Fatima Allouche, Amani Direm, Kawther Soudani

**Affiliations:** a Laboratoire des Structures, Propriétés et Interactions Inter Atomiques (LASPI^2^A), Centre Universitaire de Khenchela, 40000 Khenchela, Algeria

## Abstract

The title compound, C_7_H_8_NO_2_^+^·H_2_PO_3_^−^, is formed from alternating layers of organic cations and inorganic anions stacked along the *a*-axis direction. They are associated *via* O—H⋯O, N—H⋯O and C—H⋯O hydrogen bonding, giving rise to two different *R*_2_^2^(8) graph-set motifs and generating a three-dimensional network.

## Related literature

For applications of hybrid compounds, see: Kagan *et al.* (1999 ▶); Mazeaud *et al.* (2000 ▶); Benali-Cherif, Direm *et al.* (2007 ▶). For applications of anthranilic acid derivatives, see: He *et al.* (2003 ▶); Per Wiklund *et al.* (2004 ▶); Congiu *et al.* (2005 ▶); Nittoli *et al.* (2005 ▶). For related structured, see: Bendeif *et al.* (2003 ▶, 2009 ▶); Benali-Cherif, Allouche *et al.* (2007 ▶). For graph-set theory, see: Bernstein *et al.* (1995 ▶).
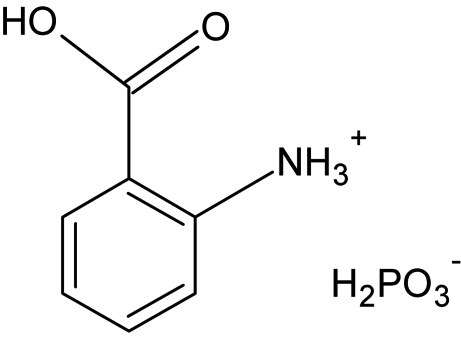


## Experimental

### 

#### Crystal data

C_7_H_8_NO_2_^+^·H_2_PO_3_^−^
*M* *_r_* = 219.13
Triclinic, 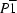

*a* = 4.8757 (6) Å
*b* = 9.4597 (6) Å
*c* = 10.0801 (5) Å
α = 78.929 (3)°
β = 76.058 (4)°
γ = 86.814 (2)°
*V* = 442.81 (7) Å^3^
*Z* = 2
Mo *K*α radiation
μ = 0.31 mm^−1^
*T* = 100 K
0.25 × 0.18 × 0.05 mm

#### Data collection

Oxford Diffraction Xcalibur Saphire2 diffractometer
Absorption correction: integration (*ABSORB*; DeTitta, 1985 ▶) *T*_min_ = 0.972, *T*_max_ = 0.985
11058 measured reflections
2581 independent reflections
2559 reflections with *I* > 2σ(*I*)
*R*_int_ = 0.035

#### Refinement

*R*[*F* ^2^ > 2σ(*F* ^2^)] = 0.033
*wR*(*F* ^2^) = 0.093
*S* = 1.07
2581 reflections
127 parameters
H-atom parameters not refined
Δρ_max_ = 0.58 e Å^−3^
Δρ_min_ = −0.24 e Å^−3^

### 

Data collection: *KappaCCD Server Software* (Nonius, 1998 ▶); cell refinement: *DENZO* and *SCALEPACK* (Otwinowski & Minor, 1997 ▶); data reduction: *DENZO* and *SCALEPACK* (Otwinowski & Minor, 1997 ▶); program(s) used to solve structure: *SIR2004* (Burla *et al.*, 2005 ▶); program(s) used to refine structure: *SHELXL97* (Sheldrick, 2008 ▶); molecular graphics: *ORTEP-3* (Farrugia, 1997 ▶) and *PLATON* (Spek, 2009 ▶); software used to prepare material for publication: *WinGX* (Farrugia, 1999 ▶).

## Supplementary Material

Crystal structure: contains datablocks global, I. DOI: 10.1107/S1600536809007077/bg2238sup1.cif

Structure factors: contains datablocks I. DOI: 10.1107/S1600536809007077/bg2238Isup2.hkl

Additional supplementary materials:  crystallographic information; 3D view; checkCIF report

## Figures and Tables

**Table 1:** Hydrogen-bond geometry (Å, °)

*D*—H⋯*A*	*D*—H	H⋯*A*	*D*⋯*A*	*D*—H⋯*A*
O1—H1⋯O3^i^	0.84	1.77	2.6085 (13)	178
N1—H1*A*⋯O4	0.91	1.96	2.8589 (14)	169
N1—H1*B*⋯O4^ii^	0.91	2.02	2.9160 (13)	169
N1—H1*C*⋯O4^iii^	0.91	1.97	2.8740 (14)	173
O5—H5*O*⋯O3^iv^	0.84	1.78	2.6059 (13)	167
C6—H6⋯O5^v^	0.95	2.55	3.2542 (15)	132

Symmetry codes: (i) 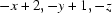; (ii) 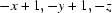; (iii) 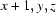; (iv) 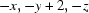; (v) 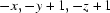.

**Fig. 1.**
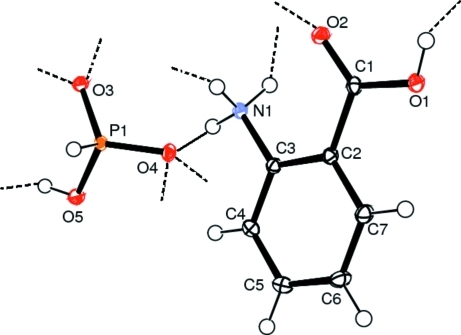
View of the asymmetric unit of C7H8NO2+.H2PO3- showing atom labels and suggesting the hydrogen bondings richness. Displacement factors drawn at a 50% level.

**Fig. 2.**
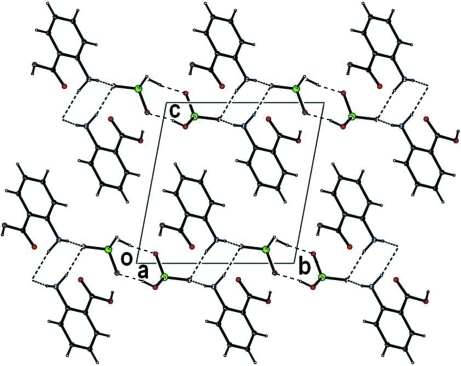
Unit cell projection down a, showing the two different R22(8) graph motifs in the structure.

## supplementary crystallographic information

### Comment

The crystal structures of organic-inorganic hybrid materials have been extensively investigated due to their interest in the field of new materials, and the number of reported structures is rapidly growing owing to their applications in medicine, material science and to their electrical, magnetic and optical properties (Kagan *et al.*, 1999; Mazeaud *et al.*, 2000) and the hydrogen bonding richness of these structures. This kind of hydrogen bonding appears in the active sites of several biological systems and is observed in similar previously studied hybrid compounds (Benali-Cherif, Direm *et al.*, 2007).

As well as being a biochemical precursor of the amino acids tryptophan, phenylalanine and tyrosine, anthranilic acid is used as a useful derivating agent for carbohydrate analysis (He *et al.*, 2003). 2-Aminobenzoic acid is present as a part of the core structure of certain alkaloids, synthetic drugs (Per Wiklund *et al.*, 2004), antiinflammatory, anticancer agents (Congiu *et al.*, 2005) and as inhibitor of Hepatitis C NS5B polymerase (Nittoli *et al.*, 2005).

The title compound structure (I) is composed of cationic HOO-C_6_H_4_—NH_3_^+^ and anionic (H_2_PO_3_^-^) groups (Fig.1). All bond lengths and angles of the (H_2_PO_3_^-^) tetrahedra and the *o*-carboxyanilinium cations are within normal ranges, in a good agreement with those observed in the litterature (Bendeif *et al.* 2003, Bendeif *et al.* 2009) and (Benali-Cherif, Allouche *et al.*, 2007), respectively.

The three H atoms of the anilinium group are subsequently involved in extensive N—H···O hydrogen-bonding (Table 1) interactions with O4 being a multiple acceptor of three different phosphite anions, while O3 behaves as double acceptor of hydrogen bonds from one cation, *via* O1 in the carboxylic group, and one anion, *via* O5 in the phosphite anion. These interactions give raise to two different *R*_2_^2^(8) graph set motifs (Bernstein *et al.* 1995), shown in  Fig. 2. In addition, there are intramolecular interactions involving the benzene ring and the carboxylic group  ensuring cohesion and stability of the crystal structiure.

### Experimental

Crystals of anthranilicium phosphite are prepared by slow evaporation at room temperature of an aqueous solution of 2-aminobenzoic acid and H_3_PO_3_ in a 1:1 stochiometric ratio.

### Refinement

The title compound crystallizes in the centrosymmetric space group P-1. A l l non-H atoms were refined with anisotropic atomic displacement parameters. All H-atoms were located in difference Fourier syntheses and refined as riding model with C—H, N—H, O—H  bond lengths constrained to 0.950 Å, 0.910 Å, 0.840Å respectively.

### Crystal data

**Table:**

C_7_H_8_NO_2_^+^·H_2_PO_3_^−^	*Z* = 2
*M**_r_* = 219.13	*F*(000) = 228
Triclinic, *P*1	*D*_x_ = 1.643 Mg m^−^^3^
Hall symbol: -P 1	Mo *K*α radiation, λ = 0.71073 Å
*a* = 4.8757 (6) Å	Cell parameters from 11058 reflections
*b* = 9.4597 (6) Å	θ = 2.8–32.7°
*c* = 10.0801 (5) Å	µ = 0.31 mm^−^^1^
α = 78.929 (3)°	*T* = 100 K
β = 76.058 (4)°	Prism, colourless
γ = 86.814 (2)°	0.25 × 0.18 × 0.05 mm
*V* = 442.81 (7) Å^3^	

### Data collection

**Table:**

Oxford Diffraction Xcalibur Saphire2 diffractometer	2581 independent reflections
Radiation source: fine-focus sealed tube	2559 reflections with *I* > 2σ(*I*)
graphite	*R*_int_ = 0.035
Detector resolution: 8.4221 pixels mm^-1^	θ_max_ = 30.0°, θ_min_ = 2.8°
ω and θ scans	*h* = −6→6
Absorption correction: integration (*ABSORB*; DeTitta, 1985)	*k* = −12→13
*T*_min_ = 0.972, *T*_max_ = 0.985	*l* = 0→14
11058 measured reflections	

### Refinement

**Table:**

Refinement on *F*^2^	Primary atom site location: structure-invariant direct methods
Least-squares matrix: full	Secondary atom site location: difference Fourier map
*R*[*F*^2^ > 2σ(*F*^2^)] = 0.033	Hydrogen site location: difference Fourier map
*wR*(*F*^2^) = 0.093	H-atom parameters not refined
*S* = 1.07	*w* = 1/[σ^2^(*F*_o_^2^) + (0.0537*P*)^2^ + 0.2002*P*] where *P* = (*F*_o_^2^ + 2*F*_c_^2^)/3
2581 reflections	(Δ/σ)_max_ < 0.001
127 parameters	Δρ_max_ = 0.58 e Å^−^^3^
0 restraints	Δρ_min_ = −0.24 e Å^−^^3^

### Special details

**Table:**

Geometry. All e.s.d.'s (except the e.s.d. in the dihedral angle between two l.s. planes) are estimated using the full covariance matrix. The cell e.s.d.'s are taken into account individually in the estimation of e.s.d.'s in distances, angles and torsion angles; correlations between e.s.d.'s in cell parameters are only used when they are defined by crystal symmetry. An approximate (isotropic) treatment of cell e.s.d.'s is used for estimating e.s.d.'s involving l.s. planes.
Refinement. Refinement of *F*^2^ against ALL reflections. The weighted *R*-factor *wR* and goodness of fit *S* are based on *F*^2^, conventional *R*-factors *R* are based on *F*, with *F* set to zero for negative *F*^2^. The threshold expression of *F*^2^ > σ(*F*^2^) is used only for calculating *R*-factors(gt) *etc*. and is not relevant to the choice of reflections for refinement. *R*-factors based on *F*^2^ are statistically about twice as large as those based on *F*, and *R*- factors based on ALL data will be even larger.

### Fractional atomic coordinates and isotropic or equivalent isotropic displacement parameters (Å^2^)

**Table:**

	*x*	*y*	*z*	*U*_iso_*/*U*_eq_	
O1	1.22190 (19)	0.11913 (10)	0.23669 (10)	0.01503 (19)	
H1	1.3765	0.1163	0.1783	0.023*	
O2	1.18445 (19)	0.33344 (10)	0.10200 (9)	0.01319 (18)	
N1	0.6956 (2)	0.48161 (11)	0.14593 (10)	0.0107 (2)	
H1A	0.5423	0.5400	0.1407	0.013*	
H1B	0.7309	0.4308	0.0754	0.013*	
H1C	0.8484	0.5361	0.1386	0.013*	
C1	1.0902 (2)	0.24260 (13)	0.20403 (12)	0.0109 (2)	
C2	0.8201 (2)	0.26393 (13)	0.30600 (12)	0.0106 (2)	
C3	0.6396 (3)	0.38167 (13)	0.27952 (12)	0.0104 (2)	
C4	0.3987 (3)	0.40572 (13)	0.37899 (13)	0.0131 (2)	
H4	0.2794	0.4862	0.3601	0.016*	
C5	0.3322 (3)	0.31144 (14)	0.50686 (13)	0.0150 (2)	
H5	0.1688	0.3284	0.5755	0.018*	
C6	0.5051 (3)	0.19272 (15)	0.53363 (13)	0.0157 (2)	
H6	0.4584	0.1276	0.6199	0.019*	
C7	0.7466 (3)	0.16969 (14)	0.43364 (13)	0.0141 (2)	
H7	0.8639	0.0883	0.4525	0.017*	
P1	0.19035 (6)	0.80587 (3)	0.08601 (3)	0.01016 (10)	
O3	0.29056 (19)	0.88933 (10)	−0.06075 (9)	0.01379 (18)	
O4	0.20062 (18)	0.64347 (9)	0.10464 (9)	0.01255 (18)	
O5	−0.1212 (2)	0.85119 (10)	0.14917 (10)	0.0166 (2)	
H5O	−0.1511	0.9363	0.1122	0.025*	
H	0.3396	0.8447	0.1629	0.050*	

### Atomic displacement parameters (Å^2^)

**Table:**

	*U*^11^	*U*^22^	*U*^33^	*U*^12^	*U*^13^	*U*^23^
O1	0.0120 (4)	0.0112 (4)	0.0180 (4)	0.0041 (3)	−0.0003 (3)	0.0013 (3)
O2	0.0124 (4)	0.0120 (4)	0.0132 (4)	0.0011 (3)	−0.0016 (3)	0.0003 (3)
N1	0.0102 (5)	0.0093 (4)	0.0118 (5)	0.0016 (3)	−0.0022 (3)	−0.0011 (3)
C1	0.0102 (5)	0.0099 (5)	0.0131 (5)	0.0011 (4)	−0.0039 (4)	−0.0025 (4)
C2	0.0097 (5)	0.0101 (5)	0.0118 (5)	0.0003 (4)	−0.0030 (4)	−0.0013 (4)
C3	0.0117 (5)	0.0093 (5)	0.0101 (5)	−0.0004 (4)	−0.0031 (4)	−0.0013 (4)
C4	0.0122 (5)	0.0128 (5)	0.0141 (5)	0.0013 (4)	−0.0020 (4)	−0.0036 (4)
C5	0.0134 (5)	0.0180 (6)	0.0128 (5)	−0.0004 (4)	−0.0006 (4)	−0.0042 (4)
C6	0.0172 (6)	0.0175 (6)	0.0103 (5)	−0.0013 (5)	−0.0016 (4)	0.0014 (4)
C7	0.0137 (5)	0.0130 (6)	0.0142 (5)	0.0016 (4)	−0.0036 (4)	0.0008 (4)
P1	0.01028 (15)	0.00778 (15)	0.01211 (15)	0.00126 (10)	−0.00279 (11)	−0.00126 (10)
O3	0.0124 (4)	0.0106 (4)	0.0148 (4)	0.0029 (3)	0.0006 (3)	0.0007 (3)
O4	0.0124 (4)	0.0082 (4)	0.0166 (4)	0.0013 (3)	−0.0040 (3)	−0.0009 (3)
O5	0.0149 (4)	0.0110 (4)	0.0181 (4)	0.0053 (3)	0.0026 (3)	0.0018 (3)

### Geometric parameters (Å, °)

**Table:**

O1—C1	1.3250 (14)	C4—H4	0.9500
O1—H1	0.8399	C5—C6	1.3902 (18)
O2—C1	1.2182 (15)	C5—H5	0.9500
N1—C3	1.4643 (15)	C6—C7	1.3908 (17)
N1—H1A	0.9100	C6—H6	0.9500
N1—H1B	0.9100	C7—H7	0.9500
N1—H1C	0.9101	P1—O4	1.5110 (9)
C1—C2	1.4930 (16)	P1—O3	1.5154 (9)
C2—C7	1.3970 (16)	P1—O5	1.5695 (9)
C2—C3	1.4060 (16)	P1—H	1.2947
C3—C4	1.3880 (16)	O5—H5O	0.8400
C4—C5	1.3969 (17)		
			
C1—O1—H1	109.5	C5—C4—H4	120.1
C3—N1—H1A	109.5	C6—C5—C4	120.00 (11)
C3—N1—H1B	109.5	C6—C5—H5	120.0
H1A—N1—H1B	109.5	C4—C5—H5	120.0
C3—N1—H1C	109.5	C5—C6—C7	119.76 (12)
H1A—N1—H1C	109.5	C5—C6—H6	120.1
H1B—N1—H1C	109.5	C7—C6—H6	120.1
O2—C1—O1	123.21 (11)	C6—C7—C2	121.25 (12)
O2—C1—C2	122.48 (11)	C6—C7—H7	119.4
O1—C1—C2	114.27 (10)	C2—C7—H7	119.4
C7—C2—C3	118.21 (11)	O4—P1—O3	116.92 (5)
C7—C2—C1	120.31 (11)	O4—P1—O5	107.62 (5)
C3—C2—C1	121.42 (11)	O3—P1—O5	109.90 (5)
C4—C3—C2	120.87 (11)	O4—P1—H	108.37
C4—C3—N1	117.68 (10)	O3—P1—H	108.24
C2—C3—N1	121.44 (10)	O5—P1—H	105.16
C3—C4—C5	119.89 (11)	P1—O5—H5O	109.5
C3—C4—H4	120.1		
			
O2—C1—C2—C7	−168.18 (12)	C2—C3—C4—C5	−0.57 (19)
O1—C1—C2—C7	9.66 (16)	N1—C3—C4—C5	178.45 (11)
O2—C1—C2—C3	9.07 (18)	C3—C4—C5—C6	−0.83 (19)
O1—C1—C2—C3	−173.09 (11)	C4—C5—C6—C7	1.1 (2)
C7—C2—C3—C4	1.67 (18)	C5—C6—C7—C2	0.1 (2)
C1—C2—C3—C4	−175.63 (11)	C3—C2—C7—C6	−1.42 (18)
C7—C2—C3—N1	−177.31 (11)	C1—C2—C7—C6	175.92 (12)
C1—C2—C3—N1	5.39 (17)		

### Hydrogen-bond geometry (Å, °)

**Table:**

*D*—H···*A*	*D*—H	H···*A*	*D*···*A*	*D*—H···*A*
O1—H1···O3^i^	0.84	1.77	2.6085 (13)	178
N1—H1A···O4	0.91	1.96	2.8589 (14)	169
N1—H1B···O4^ii^	0.91	2.02	2.9160 (13)	169
N1—H1C···O4^iii^	0.91	1.97	2.8740 (14)	173
O5—H5O···O3^iv^	0.84	1.78	2.6059 (13)	167
C6—H6···O5^v^	0.95	2.55	3.2542 (15)	132
C7—H7···O1	0.95	2.42	2.7503 (16)	101

Symmetry codes: (i) −*x*+2, −*y*+1, −*z*;  (ii) −*x*+1, −*y*+1, −*z*; (iii) *x*+1, *y*, *z*; (iv) −*x*, −*y*+2, −*z*; (v) −*x*, −*y*+1, −*z*+1.

## References

[bb1] Benali-Cherif, N., Direm, A., Allouche, F., Boukli-H-Benmenni, L. & Soudani, K. (2007). *Acta Cryst.* E**63**, o2054–o2056. 10.1107/S1600536809007077 PMC2968775 21582409

[bb2] Benali-Cherif, N., Direm, A., Allouche, F. & Soudani, K. (2007). *Acta Cryst.* E**63**, o2272–o2274. 10.1107/S1600536809007077 PMC2968775 21582409

[bb3] Bendheif, L., Benali-Cherif, N., Benguedouar, L., Bouchouit, K. & Merazig, H. (2003). *Acta Cryst.* E**59**, o141–o142.

[bb4] Bendeif, E.-E., Lecomte, C. & Dahaoui, S. (2009). *Acta Cryst.* B**65**, 59–67. 10.1107/S0108768108042298 19155560

[bb5] Bernstein, J., Davis, R. E., Shimoni, L. & Chang, N.-L. (1995). *Angew. Chem. Int. Ed. Engl.* 34, 1555–1573.

[bb6] Burla, M. C., Caliandro, R., Camalli, M., Carrozzini, B., Cascarano, G. L., De Caro, L., Giacovazzo, C., Polidori, G. & Spagna, R. (2005). *J. Appl. Cryst.* **38**, 381–388.

[bb7] Congiu, C., Cocco, M. T., Lilliu, V. & Onnis, V. (2005). *J. Med. Chem.* **48**, 8245–8252. 10.1021/jm050711d 16366606

[bb8] DeTitta, G. T. (1985). *J. Appl. Cryst.* **18**, 75–79.

[bb9] Farrugia, L. J. (1997). *J. Appl. Cryst.* **30**, 565.

[bb10] Farrugia, L. J. (1999). *J. Appl. Cryst.* **32**, 837–838.

[bb11] He, L., Sato, K., Abo, M., Okubo, A. & Yamazaki, S. (2003). *Anal. Biochem.* **314**, 128–134. 10.1016/s0003-2697(02)00633-4 12633611

[bb12] Kagan, C. R., Mitzi, D. B. C. & Dimitrakopoulos, C. D. (1999). *Science*, **286**, 945–947. 10.1126/science.286.5441.945 10542146

[bb13] Mazeaud, A., Dromzee, Y. & Thouvenot, R. (2000). *Inorg. Chem.* **39**, 4735–4740. 10.1021/ic0004784 11196948

[bb14] Nittoli, T., Curran, K., Insaf, S., DiGrandi, M., Orlowski, M., Chopra, R., Agarwal, A., Howe, A. Y. M., Prashad, A., Floyd, M. B., Johnson, B., Sutherland, A., Wheless, K., Feld, B., O’Connell, J., Mansour, T. S. & Bloom, J. (2005). *J. Med. Chem* **48**, 7560–7581. 10.1021/jm061428x 17402724

[bb15] Nonius (1998). *KappaCCD Server Software* Nonius BV, Delft, The Netherlands.

[bb16] Otwinowski, Z. & Minor, W. (1997). *Methods in Enzymology*, Vol. 276, *Macromolecular Crystallography*, Part A, edited by C. W. Carter Jr & R. M. Sweet, pp. 307–326. New York: Academic Press.

[bb17] Sheldrick, G. M. (2008). *Acta Cryst.* A**64**, 112–122. 10.1107/S0108767307043930 18156677

[bb18] Spek, A. L. (2009). *Acta Cryst.* D**65**, 148–155. 10.1107/S090744490804362X PMC2631630 19171970

[bb19] Wiklund, P. & Bergman, J. (2004). *Tetrahedron*, **45**, 969–972.

